# Experiences of violence before and during pregnancy and adverse pregnancy outcomes: An analysis of the Canadian Maternity Experiences Survey

**DOI:** 10.1186/1471-2393-11-42

**Published:** 2011-06-07

**Authors:** Marcelo L Urquia, Patricia J O'Campo, Maureen I Heaman, Patricia A Janssen, Kellie R Thiessen

**Affiliations:** 1 Centre for Research on Inner City Health, St. Michael's Hospital, 30 Bond Street, Toronto, ON M5B 1W8, Canada; 2 Dalla Lana School of Public Health, University of Toronto, 155 College Street, Health Science Building, 6th floor, Toronto, ON M5T 3M7, Canada; 3 Faculty of Nursing, University of Manitoba, 89 Curry Place, Winnipeg, MB R3T 2N2, Canada; 4 School of Population and Public Health, University of British Columbia, 2206 East Mall, Vancouver, BC V6T 1Z3, Canada

## Abstract

**Background:**

Abuse and violence against women constitute a global public health problem and are particularly important among women of reproductive age. The literature is not conclusive regarding the impact of violence against pregnant women on adverse pregnancy outcomes, such as preterm birth, small for gestational age and postpartum depression. Most studies have been conducted on relatively small samples of high-risk women. Our objective was to investigate what dimensions of violence against pregnant women were associated with preterm birth, small for gestational age and postpartum depression in a nationally representative sample of Canadian women.

**Methods:**

We analysed data of the Maternity Experiences Survey, a nationally representative survey of Canadian women giving birth in 2006. The comprehensive questionnaire included a 19-item section to collect information on different dimensions of abuse and violence, such as type, frequency, timing and perpetrator of violence. The survey design is a stratified simple random sample from the 2006 Canadian Census sampling frame. Participants were 6,421 biological mothers (78% response rate) 15 years and older who gave birth to a singleton live birth and lived with their infant at the time of the survey. Logistic regression was used to compute Odds Ratios. Survey weights were used to obtain point estimates and 95% confidence intervals were obtained with the jacknife method of variance estimation. Covariate control was informed by use of directed acyclic graphs.

**Results:**

No statistically significant associations were found for preterm birth or small for gestational age, after adjustment. Most dimensions of violence were associated with postpartum depression, particularly the combination of threats and physical violence starting before and continuing during pregnancy (Adjusted Odds Ratio = 4.1, 95% confidence interval: 1.9, 8.9) and perpetrated by the partner (4.3: 2.1, 8.7).

**Conclusions:**

Our findings provide weak evidence of an association between experiences of abuse before and during pregnancy and preterm birth and small for gestational age but they indicate that several dimensions of abuse and violence are consistently associated with postpartum depression.

## Background

Abuse and violence against women constitute an important global public health problem and women of reproductive age are at the heart of the issue [1,2]. The pregnancy period is sensitive to environmental exposures that may affect both the health of the newborn and of the mother. The reported prevalence of violence against women varies greatly between studies. Part of this heterogeneity may be due to differences in the definition and measurement of abuse, characteristics of the study populations and potential causal pathways [3,4].

The literature on violence and pregnancy outcomes is not conclusive. Most studies on newborn outcomes to date focused on low birthweight (LBW) [5-14], and preterm birth (PTB) [15-21], while only a few examined small for gestational age (or intrauterine growth restriction) [18,22]. Some studies have shown positive associations between different measures of abuse and birth outcomes [7,11,12,15,18-21] but others did not [5,9,16,17,22]. The difficulties in the interpretation of the conflicting findings on the association between abuse and LBW may be exacerbated by the fact that low birthweight is a heterogeneous indicator of two distinct, although related, processes that may lead to low birthweight, short gestation and restricted fetal growth, making it difficult to elucidate causal effects [23].

Abuse during pregnancy has also been linked to psychosocial outcomes such as high levels of stress and anxiety [24,25]. In particular, postpartum depression has also been linked to abuse [26,27].

Most studies have been conducted on women attending hospitals [7,9,18,21], community clinics [12,15] and on high-risk populations [5,8,13,17,20,22]. The relation between abuse during pregnancy and adverse pregnancy outcomes has been less frequently examined at the population-level [9,16,19]. Relatively large samples of women are needed to facilitate an inspection of the effect of different dimensions of abuse (type, timing, frequency, and perpetrators involved in abuse) on pregnancy outcomes.

The Maternity Experiences Survey (MES) provides unique nationwide Canadian data to examine different aspects of the relationship between violence against women in reproductive age and pregnancy outcomes. Our objective was to assess what dimensions of violence against pregnant women were associated with preterm birth, small for gestational age and postpartum depression in a nationally representative sample of Canadian women.

## Methods

### Study population

The Maternity Experiences Survey is a population-based survey conducted by Statistics Canada between October 23, 2006, and January 31, 2007 on behalf of the Canadian Perinatal Surveillance System, Public Health Agency of Canada. The overarching goal of the MES was to fill knowledge gaps by collecting comprehensive information on practices, factors, and maternal experiences before, during and after pregnancy [28-30].

The MES target population consisted of biological mothers who were 15 years of age and older at the time of their babies' singleton live birth in Canada, and who also lived with their infants at the time of the survey [28]. A stratified simple random sample was selected without replacement, using recent births drawn from the Census 2006 sampling frame. The sample was stratified on province or territory in which the mother resided at the time of the census and on maternal age (<20 years, > 20 years). Among 8,542 women selected from the census frame, the screening process for determining eligibility (i.e., did not have the baby in Canada or were not living with the baby by the time of the interview) was completed during data collection for 6,937 women, of which 234 were determined to be out-of-scope, leaving 6,703 women eligible to participate in the survey. Among these, 6,421 women who completed the questionnaire between five and fourteen months postpartum and gave Statistics Canada permission to provide their information to the Public Health Agency of Canada were classified as respondents [30]. The number of eligible cases was estimated by applying the proportion of eligible cases among those screened (96%) to the unscreened, resulting in an estimated 8,244 eligible cases. It was estimated that 1,541 (0.96 * 1,605) of the unscreened cases would have been eligible to participate in the survey. The number of estimated in-scope cases was therefore 8,244 (6,421 + 1,541) [29,30]. After applying the survey weights, these women represented approximately 76,500 Canadian women. The response rate was 77.9%. Compared to women who completed the questionnaires, non-respondents were more likely to be teenagers or older than 39 years, have a first language other than English or French, live in Toronto and be single. To improve the representation of the sample and reduce selection bias, total non-response was handled by adjusting the weight of individuals who responded to the survey to compensate for those who did not respond. For example, the proportion of mothers who did not speak English or French was 15.0% for the respondents and 27.0% for the non-respondents. After the weighting adjustments, the proportion was 16.9% for the respondents compared to 17.2% for the MES frame [28]. Most questionnaires were completed in a 45 minute computer-assisted telephone interview by professional female interviewers in English, French and 13 non-official languages. In about 30 cases a telephone interview was not feasible and therefore a personal interview with a paper version of the questionnaire was used. Further details of the survey design and methods have been reported elsewhere [28-30].

### Outcome measures

Singleton preterm birth was defined as a live birth before 37 completed weeks of gestation and constructed based on self-reported gestational age.

Singleton small-for-gestational age (SGA) was defined as the sex- and gestational age-specific birthweight below the 10th percentile of a Canadian population-based reference [31] and constructed based on self-reported gestational age, infant sex and birthweight. The resulting rates of singleton PTB and SGA in the survey were consistent with national surveillance data based on birth certificates [32].

Postpartum Depression (PPD) was assessed using the Edinburgh Post-Natal Depression Scale, which was also administered by the interviewers over the telephone. The scale is a ten item screening tool to identify postpartum depression at the time of its administration [33]. A score of 13 or more out of a maximum possible of 30 was used to indicate the presence of postpartum depression. Validation studies showed that the scale can detect depression in postpartum women with 86% sensitivity and 78% specificity [34,35].

### Exposures

The MES contained a section to assess abuse and violence [36]. The questions were adapted from the Violence Against Women Survey [37] to capture abuse and violence during the childbearing year. Women were asked whether in the two years prior to the interview "a spouse or partner or anyone else" had committed "acts of physical or sexual violence" and if they (i) were threatened with being hit; (ii) had something thrown at them that could have hurt them; (iii) were pushed, grabbed or shoved in a way that could have hurt them; (iv) were slapped; (v) were kicked or bit; (vi) were hit with a weapon; (vii) were beaten; (viii) were choked; (ix) were threatened with gun or knife or had one used on her; and (x) were forced into unwanted sexual activity.

Any abuse was considered to be present as any affirmative answer to any question i to x. These questions were categorised into two groups. Threats or potential hurting acts were defined as at least one affirmative answer to questions i to iii. Physical or sexual abuse was defined as at least one affirmative answer to questions iv to x. The combination of threats and physical or sexual abuse was used as a measure of severity. Women were also asked about frequency of events, categorised here as once, 2 to 5 times, and 6 times or more, and whether the perpetrator was their partner/husband/boyfriend or not (i.e., family member, friend/acquaintance or stranger). Finally, women were asked about the timing of these abusive episodes (before, during and/or after pregnancy).

### Potential confounders

There is a vast array of factors associated with both abuse and the outcomes [4,24,38-40]. We based our choice of covariates for confounder control based on a theoretical model assisted with the use of directed acyclic graphs (DAGs) [41]. DAGs entail a set of rules aimed to identify a minimally sufficient set of covariates for confounding control when the objective is to obtain an unbiased causal effect estimate of an exposure on an outcome. DAGs are particularly appropriate to prevent overcontrol (e.g., adjusting for variables affected by the exposure) and when conventional criteria for confounding control are not met. The use of DAGs also makes explicit the investigators' assumptions about the relation between the variables.

The rationale for confounding control is as follows:

Young maternal age is associated with abuse and also with single marital status and these two are predictors of poor pregnancy outcomes [4,42]. Prevalence of abuse varies according to ethnicity [4] and so does pregnancy outcomes. Controlling for maternal age, marital status and ethnicity removes the part of the effect of abuse on pregnancy outcomes that is due to differences in the distribution of these covariates. Low socioeconomic position (SEP) is a consistent predictor of both abuse and pregnancy outcomes [4,43]. Abuse can increase stress and lead to substance use (e.g., tobacco smoking, alcohol drinking, legal and illegal drug use), either directly (through coercion by abusive partner or peer-pressure) and/or indirectly (as a mechanism to cope with the stress and anxiety produced by abuse) [5,15]. Low SEP may also lead to stress and substance use. Controlling for SEP removes from the association between abuse and pregnancy outcomes the direct and indirect effects of SEP that are not related to abuse. Thus, after further adjustment for SEP the effect estimates of abuse reflect the total independent influence of abuse on pregnancy outcomes. This influence may operate through two non-exclusive pathways. A direct path may involve trauma leading to premature labour or rupture of membranes, placental abruption or a ruptured uterus, or to exacerbation of pre-existing conditions that could trigger adverse outcomes, and an indirect path through stress and/or substance use, that may involve unwanted pregnancy, poor health care utilization, poor maternal weight gain, anemia, an unhealthy diet, sexually transmitted diseases and other infections, lower social support and psychological morbidity [1,11,44]. This general conceptual model was applied to all three outcomes. However, the analyses on postpartum depression were restricted to women who had not been diagnosed with depression or took antidepressants before pregnancy to ensure that they were not depressed at the beginning of pregnancy.

Thus, variables for confounder control were maternal age (< 20 years, 20 to 34 years and 35 years and more), marital status (single, divorced, separated versus married or common-law), and immigrations status (Canadian-born and foreign-born). Socioeconomic position (SEP) was approximated by the Low income after-tax cut-off (LICO-AT) (Above LICO-AT, at or below LICO-AT and missing) [45]. The LICO-AT reflects whether the respondent lived in a household spending 20 percentage points more of their after-tax income than the average family on food, shelter and clothing, thus leaving less income available for other expenses such as health, education, transportation and recreation. The low income after-tax cut-offs are set at after-tax income levels, differentiated by size of family and area of residence.

### Analytic methods

The MES is based upon a sample design involving stratification and unequal probabilities of selection of respondents. Survey weights were used to account for the unequal probabilities of selection of respondents and thus obtain unbiased point estimates representative of the Canadian population. However, because the stratification of the sample's design affects the variance estimates calculated using the sampling weights alone, replicate methods were used to calculate variance estimates. Proportions and Odds Ratios (with 95% confidence intervals) were weighted and calculated with the jackknife method of variance estimation [46].

Frequency of violence was entered in the models as a continuous variable and therefore its Odds Ratios are interpreted as the change in the odds of the event associated with one unit increase in the number of reported episodes of violence.

Missing data were very low for most variables and therefore were not considered in the analyses, with the exception of low income, for which we created a category labelled "Missing" to prevent a significant drop in the sample size.

The data were analysed at the Toronto regional Statistics Canada Research Data Centre. All analyses were conducted with SAS 9.2^® ^(SAS Institute Inc., Cary, NC). The MES reporting guidelines prohibit the reporting of estimates based on counts less than 5 and recommend the reporting of weighted counts rounded to the nearest 100. The degree of sampling error affecting estimates was based on the coefficient of variation (CV). Estimates with a CV in excess of 33.3% are considered unreliable and therefore not reported [28].

## Results

While approximately 8400 women reported any violence in the two-year period preceding the postpartum interview (10.9%), only 3.3% were exposed during pregnancy. Table 1 shows the characteristics of the sample according to whether they were victims of abuse and violence or not. Compared with non-abused women, abused women were more likely to have depression in the postpartum period, be single, teenagers, non-immigrants, live in low income households and have a history of depression before pregnancy. Among abused women, threats or potential hurting acts were the most common type of violence and there were substantial overlap in the types of violence. Most women who suffered physical or sexual abuse also experienced threats or potential hurting acts. More than half of abused women had more than one experience of abuse. There was little overlap between violent acts perpetrated by the partner and other persons. In about one in three victims the violence occurred during pregnancy. Among these women, violence had started before pregnancy in half of them and continued during pregnancy.

**Table 1:** Characteristics of non-abused and abused women, and dimensions of abuse

Characteristic	Non-abused Weighted N = 68100	Any Abuse Weighted N = 8400
	
	Percent	Percent
**Preterm delivery**	6.1	6.6
**Small for Gestational Age**	8.3	8.2
**Postpartum depression**	6.4	15.8
**Maternal Age**		
35+ years	18.5	10.2
20 to 34 years	79.5	78.8
<20 years	2.0	11.0
**Single/divorced/separated/widowed**	6.1	26.9
**Foreign-born**	25.6	11.9
**Low income after-tax cut-off (LICO-AT)**		
Above	74.7	55.1
At or below	16.3	35.4
Missing	9.0	9.4
**Diagnosed with depression or took antidepressant before pregnancy**	14.2	25.9
**Abuse items**		
Type of abuse*		
Threats or potential hurting acts		87.3
Physical or sexual		53.8
Both combined		41.1
**Frequency of abuse**		
Once		43.4
1 to 5		37.3
6 or more		19.3
Perpretrator of abuse*		
Partner/husband/boyfriend		52.0
Family/friend/stranger/other		49.4
Both combined		1.4
Timing of abuse*		
During pregnancy		30.4
Before and during pregnancy		15.6

*Categories are not mutually exclusive and therefore do not add up to 100%.

Table 2 presents the unadjusted and adjusted associations of abuse and the outcomes. The unadjusted associations of different dimensions of abuse with preterm birth were generally positive and were stronger when the exposure started before pregnancy. Repeated physical and sexual abuse and its combination with threats or potentially harmful acts before and during pregnancy were associated with higher odds of preterm birth (OR = 2.1, 95% CI: 1.0, 4.3 and OR = 2.2, 95% CI: 1.0, 4.8, respectively). Intimate partner violence starting before pregnancy was also of borderline significance (OR = 2.1, 95% CI: 0.9, 5.0). However, these associations were no longer statistically significant after adjustment for potential confounders. Similar patterns were observed for small for gestational age, although attenuated.

**Table 2:** Odds ratios (and 95% confidence intervals) for pregnancy outcomes associated with different measures of abuse before and during pregnancy (1)

	**Preterm birth**		**Small for gestational age**		**Postpartum depression (2)**	
	**Unadjusted**	Adjusted*	**Unadjusted**	Adjusted*	**Unadjusted**	Adjusted*
	
	**OR (95% CI)**	**OR (95% CI)**	**OR (95% CI)**	**OR (95% CI)**	**OR (95% CI)**	**OR (95% CI)**
	
**Any abuse, any timing**	1.1 (0.8, 1.5)	1.0 (0.7, 1.5)	1.0 (0.7, 1.3)	0.9 (0.7, 1.3)	2.3 (1.7, 3.2)	2.5 (1.7, 3.5)
**Any abuse during pregnancy**	1.3 (0.7, 1.8)	1.2 (0.6, 2.1)	1.0 (0.6, 1.8)	1.0 (0.6, 1.7)	2.5 (1.4, 4.2)	2.6 (1.5, 4.6)
**Any abuse before and during pregnancy**	1.6 (0.8, 3.5)	1.4 (0.6, 3.2)	1.3 (0.6, 2.8)	1.2 (0.6, 2.6)	3.2 (1.6, 6.2)	3.4 (1.7, 6.8)
						
**Abuse items**						
**By type, during pregnancy**						
Could have hurt or threats	1.1 (0.5, 2.1)	1.0 (0.5, 2.0)	1.1 (0.6, 1.9)	1.0 (0.6, 1.9)	2.4 (1.4, 4.2)	2.2 (1.2, 4.1)
Physical or sexual	1.8 (0.9, 3.4)	1.7 (0.8, 3.3)	1.0 (0.5, 2.1)	1.0 (0.5, 2.0)	2.6 (1.4, 4.8)	2.3 (1.2, 4.4)
Both combined	1.5 (0.7, 3.3)	1.3 (0.6, 3.1)	1.1 (0.5, 2.3)	1.0 (0.4, 2.2)	3.1 (1.6, 6.0)	3.3 (1.6, 6.7)
						
**By type, before and during pregnancy**						
Could have hurt or threats	1.7 (0.7, 3.8)	1.5 (0.6, 3.7)	1.4 (0.6, 3.0)	1.3 (0.6, 2.8)	3.2 (1.6, 6.4)	2.8 (1.4, 5.7)
Physical or sexual	2.1 (1.0, 4.5)	1.9 (0.8, 4.3)	1.5 (0.7, 3.2)	1.4 (0.6, 3.1)	3.1 (1.5, 6.2)	2.7 (1.3, 5.7)
Both combined	2.2 (1.0, 5.0)	1.9 (0.8, 4.7)	1.5 (0.7, 3.5)	1.3 (0.6, 3.2)	3.9 (1.9, 7.9)	4.1 (1.9, 8.9)
						
**By frequency, during pregnancy**						
Per 1 additional episode	1.1 (1.0, 1.2)	1.0 (0.9, 1.2)	1.0 (0.9, 1.1)	1.0 (0.9, 1.1)	1.2 (1.1, 1.3)	1.2 (1.1, 1.3)
						
**By frequency, before and during pregnancy**						
Per 1 additional episode	1.1 (1.0, 1.2)	1.0 (0.9, 1.2)	1.0 (0.9, 1.1)	1.0 (0.9, 1.1)	1.2 (1.1, 1.2)	1.2 (1.1, 1.3)
						
**By perpetrator, during pregnancy**						
Partner/husband/boyfriend	1.1 (0.5, 2.5)	0.9 (0.4, 2.2)	1.2 (0.6, 2.5)	1.1 (0.5, 2.3)	3.9 (2.4, 6.5)	3.8 (2.2, 6.7)
Family/friend/stranger/other	1.4 (0.6, 3.0)	1.3 (0.6, 2.9)	0.8 (0.3, 2.0)	1.0 (0.5, 2.2)	1.4 (0.7, 2.8)	1.6 (0.8, 3.0)
						
**By perpetrator, before and during pregnancy**						
Partner/husband/boyfriend	2.1 (0.9, 5.0)	1.8 (0.7, 4.3)	2.1 (0.9, 4.8)	1.8 (0.8, 4.4)	4.5 (2.4, 8.4)	4.3 (2.1, 8.7)
Family/friend/stranger/other	Not reliable	Not reliable	Not reliable	Not reliable	Not reliable	Not reliable

(1) For all comparisons, the reference group is composed of non-abused women

(2) Restricted to women who were not diagnosed with depression before pregnancy or did not take antidepressants before pregnancy

* Adjusted for maternal age groups, marital status, immigration status, and living in a low income household

Most of the dimensions of abuse were associated with postpartum depression. The associations became stronger when abusive acts occurred both before and during pregnancy and when threats or potentially harmful acts were combined with physical and sexual abuse. The odds of postpartum depression increased 20% with each additional episode of violence. Violence inflicted by the partner was also strongly associated with depression but not abusive acts perpetrated by other persons. Abuse starting before pregnancy and perpetrated by persons other than the partner produced unreliable estimates and therefore could not be reported.

## Discussion

In a nationally representative sample of Canadian women, different measures of abuse during pregnancy were marginally associated with preterm birth and small for gestational age after adjustment for potential confounders but they were consistently associated with postpartum depression. The strength of the associations increased with the severity and chronicity of abuse. Associations were stronger when violent events were recurrent, when abusive episodes included both physical abuse and threats and potential hurting acts, took place before and during pregnancy and when the intimate partner was the perpetrator.

### Strengths and limitations

Our study has some strengths. This national population-based survey yielded one of the largest samples to date in the investigation of the associations of interest. Its comprehensive questionnaire assessed several dimensions of abuse (perpetrators, frequency, timing and type) and collected information on a broad set of covariates. We based our analyses on explicit conceptual models based on previous research and causal theory.

Among the limitations of our study, we can first mention the cross-sectional survey design. Information was self-reported and most exposures and outcomes, excepting postpartum depression, were retrospectively assessed in the postpartum interview and therefore may be somewhat affected by recall bias.

The method used to collect information in the survey, computer assisted telephone interview (CATI), has been found to produce higher response rates than other methods such as Interactive Voice Response (IVR) or Web-based questionnaires but lower proportions of self-reported socially undesirable characteristics [47], although that was not a consistent finding [48,49]. In particular, one study did not find substantial differences in the proportions of physical and sexual abuse reported according to IVR and CATI [49]. As the efficacy of the CATI may be influenced by the experience of the interviewers [50], it is unlikely that the MES was negatively affected by an incorrect application of the method since highly skilled interviewers were employed, with extensive training and observation by senior interviewers [28]. If the application of CATI underestimated the prevalence of abuse in the MES, the literature suggests that the resulting bias would be small.

Although the questionnaire was quite comprehensive, it lacked key questions about lifetime abuse and whether violent episodes resulted in physical harm. Control for obstetric history was not considered because prior adverse outcomes might have also been influenced by abuse in the past. But, as such information was not available in the survey, residual confounding by obstetric history cannot be ruled out [51]. Although the sample size was relatively large for this kind of study, the low prevalence of the exposure during pregnancy may have contributed to the failure to detect moderate statistically significant associations with preterm birth and small for gestational age. Finally, the efficiency of adjusting for socioeconomic position in the full models may be low since this variable was measured at the individual-level, and neighbourhood deprivation may play an important role in the relationships of interest [52].

### Comparison with other studies

Our results regarding preterm birth and small for gestational age are not at odds with previous findings. The majority of studies focused on low birthweight rates, which reflect a balance between rates of short gestation and fetal growth restriction. As in our findings, these studies found positive associations or null associations but no study to date has reported a protective effect of violence on pregnancy outcomes. Indeed, in one meta-analysis assessing abuse as a risk factor for low birthweight that included eight studies, seven of which reported non-statistically significant associations, the pooled odds ratio was 1.4 (96% confidence interval: 1.1, 1.8) [11].

We also found a trend towards severity and chronicity, which is consistent with previous findings. Among studies reporting positive associations were those in which the definition of violence included more severe cases, such as injuries resulting from abuse [18,20] or episodes of violence reported to police [19].

Our results regarding postpartum depression are also consistent with previous literature linking it to abuse [1,25,26]. Beydoun and colleagues [26], also using the Maternity Experiences Survey, found that intimate partner violence was associated with postpartum depression after adjustment for covariates (Odds Ratio = 1.61, 95% confidence interval: 1.06, 2.45). However, our results show stronger associations, mainly because we excluded women with a history of depression before pregnancy, assessed different dimensions of violence and did not adjust for potential mediators, such as unwanted pregnancy and health behaviours, which are likely to be affected by abuse.

### Interpretation

Although our findings provide weak evidence of an association between experiences of abuse and preterm birth and small for gestational age, they should not be interpreted as evidence of an absence of association. Although the point estimates of the odds ratios were within the range reported in the studies showing positive associations between abuse and preterm birth and low birthweight [11,15,19-21], our ability to identify significant associations according to a 5% significance level criterion was limited by the relatively low prevalence of abuse during pregnancy in the sample. Although the prevalence of abuse in the sample was 10.9%, only 3.3% occurred during pregnancy, which is the critical exposure window for preterm birth and small for gestational age. However, interpretation of the confidence intervals in terms of their clinical significance suggests the presence of positive associations [53], particularly for preterm birth. The observed associations between abuse and birth outcomes may have been underestimated due to additional reasons. First, the questionnaire did not include questions to assess if the reported abuse resulted in different degrees of physical harm, which may reveal a more distinct physical injury pathway [18-20]. Second, the sample did not include women whose babies died in the period from birth to interview. If some of these neonatal and infant deaths were related to the most serious battering during pregnancy [18,19,54], their exclusion may partially account for the dilution of effects. Third, it is possible that the associations between violence and birth outcomes are more evident in hospital-based studies [7,18,21] and high-risk samples [13,20] compared with the diverse sample at the Canadian population level. In addition, clinic-based studies tend to show higher rates of intimate partner violence than population based studies. Finally, small for gestational age is perhaps the outcome most affected by poor recall, since it is based on the combination of self-reported gestational age and birthweight. By definition, SGA infants should comprise 10% of the population [31] but in the MES survey was 8.3%. The drop in the rate of SGA is explained by the secular increase in the size of live births in Canada [32,55], since the birthweight reference was based on infants born in the years 1994 to 1996, when babies were somewhat smaller [31]. It is unlikely, however, that the smaller rate of SGA could account for the failure to detect associations with violence during pregnancy in our study.

The findings on postpartum depression provide stronger evidence of a causal association. Although the design was cross-sectional, the survey included detailed information on the timing when the abuse occurred. Restriction of the exposure to the pregnancy period ensured that the exposure preceded the outcome. Moreover, we excluded women with a history of depression prior to pregnancy thus creating a retrospective cohort of women free of the condition at the time of the exposure and therefore ruling out confounding by chronic depressive symptoms.

## Conclusions

Overall, our findings provide weak evidence of an association between experiences of abuse during pregnancy and preterm birth and small for gestational age but indicate that most dimensions of abuse during pregnancy are consistently associated with postpartum depression. Violence against women in general, and of reproductive age in particular, is a negative outcome in itself and therefore its prevention is warranted regardless its association with adverse health outcomes. Our findings further highlight the importance of the prevention of violence against childbearing women based on the negative impact on women's postpartum mental health.

## Competing interests

The authors declare that they have no competing interests.

## Authors' contributions

MU designed the study, analysed the data and led the writing of the manuscript. PO, MH and PJ contributed to the design and interpretation of results. KT conducted the literature search. All authors revised and approved the manuscript.

## Pre-publication history

The pre-publication history for this paper can be accessed here:

http://www.biomedcentral.com/1471-2393/11/42/prepub
